# Improving Surfactin Production in *Bacillus subtilis* 168 by Metabolic Engineering

**DOI:** 10.3390/microorganisms12050998

**Published:** 2024-05-15

**Authors:** Zihao Guo, Jiuyu Sun, Qinyuan Ma, Mengqi Li, Yamin Dou, Shaomei Yang, Xiuzhen Gao

**Affiliations:** School of Life Sciences and Medicine, Shandong University of Technology, 266 Xincun West Road, Zibo 255049, China; m17658591621@163.com (Z.G.); sun2168099487@outlook.com (J.S.); qyma@sdut.edu.cn (Q.M.); mengqi_0510@163.com (M.L.); 18295496869@163.com (Y.D.)

**Keywords:** surfactin, *Bacillus subtilis*, phosphopantetheinyl transferase, surfactin transporter, global transcriptional regulator

## Abstract

Surfactin is widely used in the petroleum extraction, cosmetics, biopharmaceuticals and agriculture industries. It possesses antibacterial and antiviral activities and can reduce interfacial tension. *Bacillus* are commonly used as production chassis, but wild-type *Bacillus subtilis* 168 cannot synthesise surfactin. In this study, the phosphopantetheinyl transferase (PPTase) gene sfp* (with a T base removed) was overexpressed and enzyme activity was restored, enabling *B. subtilis* 168 to synthesise surfactin with a yield of 747.5 ± 6.5 mg/L. Knocking out *ppsD* and *yvkC* did not enhance surfactin synthesis. Overexpression of predicted surfactin transporter gene *yfiS* increased its titre to 1060.7 ± 89.4 mg/L, while overexpression of *yerP*, *ycxA* and *ycxA-efp* had little or negative effects on surfactin synthesis, suggesting YfiS is involved in surfactin efflux. By replacing the native promoter of the *srfA* operon encoding surfactin synthase with three promoters, surfactin synthesis was significantly reduced. However, knockout of the global transcriptional regulator gene *codY* enhanced the surfactin titre to 1601.8 ± 91.9 mg/L. The highest surfactin titre reached 3.89 ± 0.07 g/L, with the yield of 0.63 ± 0.02 g/g DCW, after 36 h of fed-batch fermentation in 5 L fermenter. This study provides a reference for further understanding surfactin synthesis and constructing microbial cell factories.

## 1. Introduction

Surfactin is a lipopeptide biosurfactant with a cyclic structure composed of seven amino acid residues (both L- and D-amino acids) connected to a long β-hydroxyl fatty acid chain (usually C13−C15) by a lactone bond, making it an amphiphilic molecule [1,2,3]. Due to its strong ability to reduce interfacial tension between oil and water, surfactin is used to reduce the emulsification and viscosity of crude oil for enhanced oil recovery in the petroleum extraction industry [4,5,6]. Moreover, it can be effectively applied in cosmetics in the form of solvents, soaps and facial cleansers due to its emulsification, washing, foaming, solubilisation, wetting, penetration, dispersion and low toxicity characteristics [7,8]. The long-chain fatty acid of surfactin can penetrate into the interior of cell membranes, causing dissolution and damage, which is the basis for its antibacterial activity [9,10]. Surfactin can also break down viral lipid membranes, and it has antiviral properties [11]. Therefore, it can be applied as a biological preservative to effectively control the growth of harmful microorganisms in food [12], and it may prevent fungal infections and skin diseases [13]. In addition, it has broad application prospects in the agricultural field [14], such as controlling apple scab [15].

Currently, the main production methods for surfactin include chemical synthesis and microbial fermentation, with the latter favoured due to superior environmental friendliness and biocompatibility. However, low productivity of surfactin remains an important limiting factor in its industrial production [16,17]. For instance, combinatorial metabolic engineering of *Bacillus subtilis* ATCC 21332, including enhanced nitrate reduction, fatty acid hydroxylation, rational transporter engineering and feeding, yielded 14.4 g/L of surfactin, with the productivity of 0.6 g/L/h, the highest reported productivity to date [18]. Therefore, the development of microbial cell factories is crucial for its overproduction. Surfactin was first discovered in the fermentation broth of *B*. *subtilis* in 1968, with four isomers identified [19]. Moreover, *B. subtilis*, a model Gram-positive bacterium, grows fast and is easy to cultivate. It has excellent expression systems with good genetic stability, excellent protein secretion ability, no strong codon preferences and mature gene modification technologies. It has been recognised by the USA Food and Drug Administration (FDA) as a ‘Generally Recognised as Safe’ (GRAS) strain [20]. Therefore, *B. subtilis* is commonly used as a chassis for producing surfactin through metabolic engineering [18,21,22].

**Figure 1:**
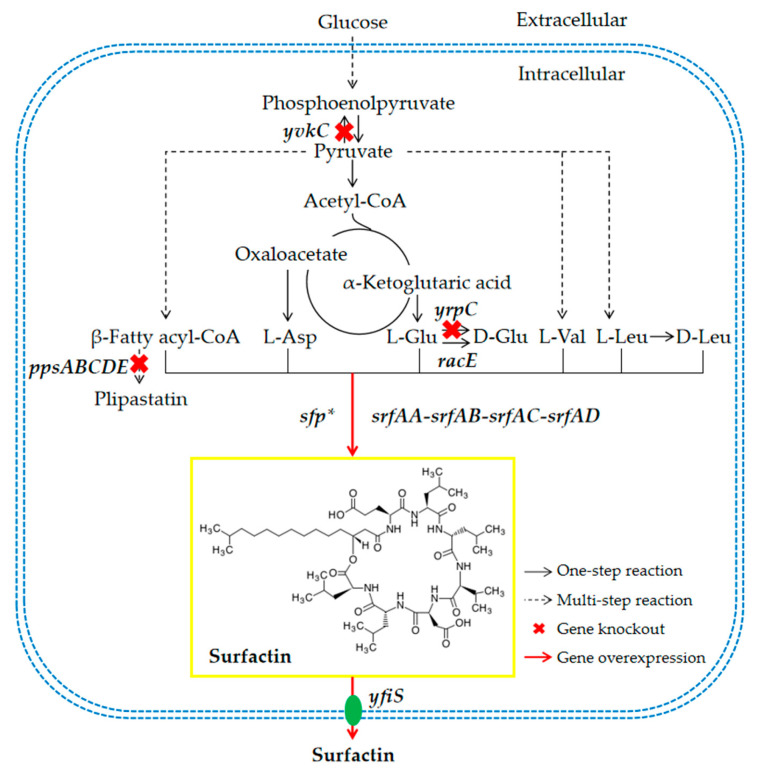
Schematic diagram of the biosynthetic pathway and the overall engineering strategy of surfactin in *Bacillus subtilis*. *yvkC*, gene encoding phosphotransferase; *ppsABCDE*, the operon encoding plipastatin synthetase; *yrpC*, gene encoding L-glutamate racemase (non-essential gene); *racE*, gene encoding L-glutamate racemase (essential gene); sfp*, gene encoding active phosphopantetheinyl transferase; *srfAA-srfAB-srfAC-srfAD*, the operon encoding surfactin synthetase; *yfiS*, gene encoding the surfactin transporter.

In *B. subtilis*, the biosynthesis of surfactin mainly involves the synthesis and structural assembly of precursors (fatty acids and amino acids). *B. subtilis* synthesises the corresponding precursors through primary metabolism, such as glycolysis, the tricarboxylic acid cycle, protein metabolism, lipid metabolism and other pathways (Figure 1). The assembly of surfactin is then catalysed by a non-ribosomal peptide synthase (NRPS) system, which includes initiation, peptide chain extension, cyclisation and release [23,24]. The NRPS, also known as surfactin synthase, is a multi-domain protein composed of multiple modules, including the SrfAA, SrfAB, SrfAC and SrfAD subunits. They are encoded by *srfAA*, *srfAB*, *srfAC* and *srfAD*, respectively, together forming the 27 kb *srfA* operon [24]. Transcription of the *srfA* operon is regulated by the quorum sensing system ComQXPA, and phosphorylated ComA binds to a specific region of its promoter, activating its transcription [25,26,27]. In addition, downstream of the *srfA* operon, there is a phosphopantetheinyl transferase (PPTase) gene *sfp* that is essential for surfactin synthesis, which functions to activate surfactin synthase [28]. However, *sfp* is an inactive pseudogene in wild-type *B. subtilis* 168, which cannot synthesise surfactin. Wu et al., (2019) integrated the *sfp* gene from *Bacillus amyloliquefaciens* MT45 into the genome of *B. subtilis* 168 at the same locus, resulting in a surfactin titre of 400 mg/L [21]. Subsequently, biofilm formation-related genes were knocked out, potential self-resistance associated proteins were overexpressed, the branched-chain fatty acid biosynthesis pathway was engineered, *comQXPA* from *B. amyloliquefaciens* MT45 was overexpressed and the global transcriptional regulator gene *codY* was knocked out, resulting in a surfactin titre of 12.8 g/L [21]. Wang et al., (2019) integrated *sfp* from *B. amyloliquefaciens* DSM7 into the *ydeO* (encoding an unknown protein) locus of the genome of *B. subtilis* 168, resulting in a surfactin titre of 450 mg/L [29]. The clustered regularly interspersed short palindromic repeat interference (CRISPRi) technology was used to inhibit the expression of L-glutamate racemase genes *yrpC* and *racE*, resulting in a surfactin titre of 750 mg/L [29]. Hu et al., (2020) integrated *sfp* from *Bacillus velezensis* BS-37 into the genome of *B. subtilis* 168 at the same locus, resulting in a surfactin titre of 982 ± 98 mg/L [30]. Medium-chain acyl-acyl carrier protein (ACP) thioesterase (encoded by *bte*) and fatty acyl CoA ligase (encoded by *yhfL*) were overexpressed, and acyl-CoA dehydrogenase gene *fadE* was knocked out, resulting in a surfactin titre of 2203 mg/L, with xylose as the carbon source [30]. However, there have been no studies on the overexpression of endogenous active *sfp* in *B. subtilis* 168. In addition, the efflux of surfactin is an important factor limiting its synthesis. Overexpression of three putative lipopeptide transporters genes: *yerP* (endogenous), *ycxA* and *krsE* (heterologous) in *B. subtilis* THY-7 resulted in a 145%, 89% and 52% increase in surfactin production, respectively [31], and overexpression of endogenous *yerP* in *B. subtilis* 168 resulted in a 35.3% increase in surfactin production [21], demonstrating that YerP acts as the major surfactin exporter. Research by Xia et al., (2024) showed that preprotein translocase SecA, signal recognition particle receptor FtsY and cell division ATP-binding protein FtsE are also involved in the transport of surfactin in *B. subtilis* ATCC 21332 [18].

The present study used *B. subtilis* 168 as the parent strain. To facilitate gene knockout or integrated expression [32], strain BS168N integrated with P*_ara_*-*neo* on the genome was employed. Firstly, the excess T base of endogenous *sfp* was removed, and the modified gene was integrated into the genome of BS168N for overexpression. The resulting recombinant strain was fermented and surfactin synthesis assessed. Next, plipastatin synthase gene *ppsD* and phosphotransferase gene *yvkC* were knocked out to reduce the consumption of amino acids, hydroxy fatty acids and ATP, and their effects on surfactin synthesis were investigated. Thirdly, endogenous *yerP*, *ycxA* and predicted surfactin transporter gene *yfiS* were integrated into the same locus of the genome for overexpression, and their effects on surfactin secretion were evaluated. Fourthly, the native promoter of the *srfA* operon was replaced in situ by constitutive promoters, and *codY* was knocked out, so their impacts on the transcriptional level of the *srfA* operon and surfactin synthesis were explored. Finally, the final recombinant strain was subjected to fed-batch fermentation, and strain growth and surfactin production were measured.

## 2. Materials and Methods

### 2.1. Microorganisms, Media and Culture Conditions

*Bacillus subtilis* 168 (BS168) and BS168N were provided by Tianjin University. All recombinant *B. subtilis* strains were constructed based on BS168N. The strains and plasmids used in this study are listed in Table 1. Luria–Bertani (LB) medium (10 g/L tryptone, 5 g/L yeast extract and 10 g/L NaCl) was used for strain cultivation. After adding the fragments to the competent cells, they were coated on LBC8 solid plates (added with 15 g/L of agar and 8 μg/mL of chloramphenicol to LB liquid medium) for preliminary screening of the transformants. After culturing the transformants in LB liquid medium for 4 h, they were coated on LBN16 solid plates (added with 15 g/L of agar and 16 μg/mL of neomycin to LB liquid medium) for secondary screening of the recombinant bacteria. A medium (50 mL, containing 25 g/L glucose, 10 g/L tryptone, 3 g/L beef extract, 1 g/L KH_2_PO_4_, 1 g/L K_2_HPO_4_, 1 g/L NaCl, 0.5 g/L (NH_4_)_2_SO_4_, 0.2 g/L L-leucine, 0.05 g/L sodium glutamate, 0.05 g/L MgSO_4_·7H_2_O, 0.05 g/L MnSO_4_·H_2_O and 7.5 mg/L FeSO_4_·7H_2_O, pH 7.0) was used for the fermentation experiments. Each *B. subtilis* strain was transferred into a 250 mL flask with 30 mL LB liquid medium and incubated at 37 °C with shaking at 200 rpm for 12 h as the seed culture. Next, 2.5 mL of seed culture was inoculated into 250 mL flasks containing 50 mL fermentation medium and incubated at 37 °C with shaking at 220 rpm for 48 h.

### 2.2. Construction of B. subtilis Knockout Mutants

For the knockout of *yrpC*, the 5039 bp UDCRG fragment was amplified from the genome of BSD1-ΔyrpCm [33] using primer pair yrpC-U1/yrpC-G2 (Table 2). U refers to the upstream homologous arm, G refers to the downstream homologous arm, D refers to the homologous recombinant fragment, C refers to the fragment containing the chloramphenicol resistance gene *cat* and R refers to the fragment containing gene *araR* encoding the transcriptional repressor of the arabinose operon [32]. AraR could inhibit the transcription of P*_ara_*-*neo*, resulting in the strain not exhibiting neomycin resistance but exhibiting chloramphenicol resistance [32]. This UDCRG fragment was used to transform competent cells of BS168N as previously described [35], and the cells were cultured on chloramphenicol resistance plates. The *yrpC* knockout strain BSSF1 was obtained by a two-step screening process as previously described [32].

For the knockout of *ppsD*, 1101 bp U, 1089 bp D and 919 bp G fragments were amplified from the genome of BS168 using primer pairs ppsD-U1/ppsD-U2, ppsD-D1q/ppsD-D2 and ppsD-G1q/ppsD-G2, respectively. A 2136 bp cat-araR (CR) fragment was amplified from BSD1-ΔyrpCm using primer pair ppsD-CR1q/ppsD-CR2. These four fragments were spliced in the order of U-D-CR-G by two rounds of overlap extension PCR (OE-PCR) using primer pairs ppsD-U1/ppsD-D2 and ppsD-U1/ppsD-G2, respectively. Finally, this UDCRG fragment was used to transform competent cells of BSSF2. The resulting *ppsD* knockout strain BSSF3 was obtained by a two-step screening process.

For the knockout of *yvkC*, the 5259 bp UDCRG fragment was amplified from the genome of BSD1-ΔyvkCm [33] using primer pair yvkC-U1/yvkC-G2. This UDCRG fragment was used to transform competent cells of BSSF3. The resulting *yrpC* knockout strain BSSF4 was obtained by a two-step screening process.

For the knockout of *codY*, the 1116 bp U, 1092 bp D and 605 bp G fragments were amplified from the genome of BS168 using primer pairs codY-U1/codY-U2, codY-D1q/codY-D2 and codY-G1q/codY-G2, respectively. A 2189 bp CR fragment was amplified from BSD1-ΔyrpCm using primer pair codY-CR1q/codY-CR2. These four fragments were spliced in the order of U-D-CR-G by two rounds of OE-PCR. Finally, this UDCRG fragment was used to transform competent cells of BSSF52. The resulting *codY* knockout strain BSSF64 was obtained by a two-step screening process.

### 2.3. Construction of B. subtilis Overexpression Mutants

For overexpression of sfp*, 1062 bp U and 507 bp S1 fragments containing the partial nucleotide sequence of *sfp*, and the 204 bp S2 fragment containing the residual nucleotide sequence of sfp, were amplified from the genome of *B. subtilis* 168 using primer pairs yrpC-U1/yrpC-U2q, sfp1-1q/sfp1-2 and sfp2-1/sfp2-2, respectively. A 183 bp P fragment containing the constitutive promoter TP2 expression cassette [36] was amplified from plasmid pUC57-simple-VHb [34] using primer pair TP2-1/TP2-2. A 3978 bp DCRG fragment was amplified from BSD1-ΔyrpCm using primer pair yrpC-D1q/yrpC-G2. Fragments P, S1 and S2 were then spliced in the order of P-S1-S2 using primer pair TP2-1/sfp2-2, and fragments U, PS1S2 and DCRG were spliced in the order of U-PS1S2-DCRG using primer pair yrpC-U1/yrpC-G2. Finally, this fragment was used to transform competent cells of BS168N. The resulting strain BSSF2 overexpressing sfp* was obtained by a two-step screening process.

For overexpression of *yerP*, a 1399 bp fragment of UP containing the upstream homologous arm U and the constitutive P*_43_* promoter [37], and a 3270 bp fragment of *yerP* containing the complete nucleotide sequence and terminator sequence of *yerP*, were amplified from the genome of BSΔ6-AD1m [33] using primer pairs yvkC-U1/yvkC-P2 and yerP-1q/yerP-2, respectively. A 3999 bp DCRG fragment was amplified from BSD1-ΔyvkCm using primer pair yvkC-yerP-D1q/yvkC-yerP-G2. These three fragments were spliced in the order of UP-*yerP*-DCRG using primer pair yvkC-U1/yvkC-yerP-G2. Finally, this fragment was used to transform competent cells of BSSF3. The resulting strain BSSF51 overexpressing *yerP* was obtained by a two-step screening process.

For overexpression of *yfiS*, a 1399 bp fragment of UP, a 1332 bp fragment of yfiS containing the complete nucleotide sequence of *yfiS* and a 3954 bp fragment of DCRG were amplified from the genome of BSSF51m using primer pairs yvkC-U1/yvkC-P2, yfiS-1q/yfiS-2 and yvkC-yfiS-D1q/yvkC-yfiS-G2, respectively. These three fragments were spliced in the order of UP-*yfiS*-DCRG using primer pair yvkC-U1/yvkC-yfiS-G2. Finally, this fragment was used to transform competent cells of BSSF3. The resulting strain BSSF52 overexpressing *yfiS* was obtained by a two-step screening process.

For overexpression of *ycxA*, a 1399 bp fragment of UP, a 1489 bp fragment of *ycxA* containing the complete nucleotide sequence and terminator sequence of *ycxA* and a 3847 bp fragment of DCRG were amplified from the genome of BSSF51m using primer pairs yvkC-U1/yvkC-P2, ycxA-1q/ycxA-2 and yvkC-ycxA-D1q/yvkC-yfiS-G2, respectively. These three fragments were spliced in the order of UP-*ycxA*-DCRG using primer pair yvkC-U1/yvkC-yfiS-G2. Finally, this fragment was used to transform competent cells of BSSF3. The resulting strain BSSF53 overexpressing *ycxA* was obtained by a two-step screening process.

For overexpression of *ycxA-efp*, a 2631 bp fragment of UPA containing the upstream homologous arm U, the P*_43_* promoter the complete nucleotide sequence of *ycxA*, a 689 bp fragment of *efp* containing the nucleotide sequence and terminator sequence and a 3872 bp fragment of DCRG were amplified from the genome of BSSF53m using primer pairs yvkC-U1/2-ycxA-2, efp-1q/efp-2 and yvkC-efp-D1q/yvkC-yfiS-G2, respectively. These three fragments were spliced in the order of UPA-*efp*-DCRG using primer pair yvkC-U1/yvkC-yfiS-G2. Finally, this fragment was used to transform competent cells of BSSF3. The resulting strain BSSF54 overexpressing *ycxA-efp* was obtained by a two-step screening process.

### 2.4. In Situ Substitution of the Native Promoter of the srfA Operon

The native promoter of the *srfA* operon was replaced in situ by the P*_HpaII_* promoter. First, 1346 bp U, 850 bp C and 820 bp D fragments were amplified from the genome of BSD1-ΔyrpCm using primer pairs srf-U1/srf-2, srf-C1q/srf-C2 and srf-D1q/srf-D2, respectively. A 274 bp P1 fragment containing the constitutive P*_HpaII_* promoter was amplified from plasmid pMA5 using primer pair srf-P1-1q/srf-P1-2. These four fragments were spliced in the order of U-C-P1-D using primer pair srf-U1/srf-D2. Finally, this fragment was used to transform competent cells of BSSF52. The resulting strain BSSF61 was obtained by a one-step screening process.

The native promoter of the *srfA* operon was replaced in situ by the P*_43_* promoter. First, 2196 bp UC and 820 bp D fragments were amplified from the genome of BSSF61 using primer pairs srf-U1/2-srf-C2 and 2-srf-D1q/srf-D2, respectively. A 267 bp P2 fragment containing the P*_43_* promoter was amplified from the genome of BSΔ6-AD1m using primer pair srf-P2-1q/srf-P2-2. These three fragments were spliced in the order of UC-P2-D using primer pair srf-U1/srf-D2. Finally, this fragment was used to transform competent cells of BSSF52. The resulting strain BSSF62 was obtained by a one-step screening process.

The native promoter of the *srfA* operon was replaced in situ by the P*_SB_* expression cassette. First, 2196 bp UC and 820 bp D fragments were amplified from the genome of BSSF61 using primer pairs srf-U1/2-srf-C2 and 3-srf-D1q/srf-D2, respectively. A 128 bp P3 fragment containing the constitutive promoter P*_SB_* expression cassette [38] was amplified from plasmid pUC57-simple-PyrG^E156K^ [33] using primer pair srf-P3-1q/srf-P3-2. These three fragments were spliced in the order of UC-P3-D using primer pair srf-U1/srf-D2. Finally, this fragment was used to transform competent cells of BSSF52. The resulting strain BSSF63 was obtained by a one-step screening process.

### 2.5. Extraction and Detection of Surfactin

The surfactin standard was purchased from Sigma-Aldrich (St. Louis, MO, USA). A 20 mL volume of fermentation broth was centrifuged at 8000 rpm for 10 min, and the pH of the supernatant was adjusted to 2.0 with HCl. After incubating overnight at 4 °C, centrifuging at 8000 rpm for 10 min and discarding the supernatant, the precipitate was dried at room temperature, 5 mL of methanol was added to dissolve the precipitate and the sample was filtered through a 0.22 μm organic filter. The concentration of surfactin was measured using an LC-2030 reversed-phase high-performance liquid chromatography (HPLC) system (Shimadzu, Kyoto, Japan) equipped with a ZORBAX SB-C18 column (250 mm × 4.6 mm, 5 μm; Agilent, Santa Clara, CA, USA) and a UV detector (Shimadzu) at 205 nm. The column temperature was set to 30 °C. The mobile phase consisted of methanol/water/trifluoroacetic acid solution (90:10:0.1, *v*/*v*/*v*) and the flow rate was 1.0 mL/min.

### 2.6. Quantitative Real-Time PCR (qRT-PCR)

A 1 mL volume of fermentation broth was collected after 24 h of fermentation, and the total RNA was extracted using a Bacterial Total RNA Rapid Extraction Kit (HLingene, Shanghai, China). Reverse-transcription was performed using a NG Script I cDNA Synthesis Kit (HLingene) to obtain the cDNA libraries. qRT-PCR was carried out using a LightCycler 480 instrument (Roche, Mannheim, Germany) using the 2× SYBR Green qPCR Mixture (HLingene). The relative transcriptional levels were calculated according to Pfaffl [39], with the carbon catabolite control protein A gene *ccpA* serving as an internal control.

### 2.7. Fed-Batch Fermentation

Fed-batch fermentation was conducted in a 5 L fermenter at the National Center of Bio-Engineering & Technology (Shanghai, China). A single colony of strain BSFF64 was inoculated into 5 mL LB medium and cultured at 37 °C, with shaking at 200 rpm overnight. The preculture was transferred into 100 mL fresh fermentation medium and cultured at 37 °C and 220 rpm for 12 h. Next, 100 mL seed culture was transferred to 1.9 L fermentation medium in a 5 L bioreactor, and fermentation was carried out at 37 °C with the pH maintained at 7.0 using NaOH (1 mol/L) and HCl (1 mol/L). The airflow was set at 2 vvm, with agitation at 300−500 rpm. The feed solution containing 240 g/L glucose, 30 g/L tryptone, 25 g/L beef extract, 5 g/L L-leucine and 75 mg/L FeSO4·7H2O was added automatically to maintain the dissolved oxygen (DO) at 40−50%. Duplicate samples were collected to determine the cell density and surfactin titre.

### 2.8. Statistical Analysis

Analysis of the response variables was performed using SPSS statistics 17.0 [40]. One-way analysis of variance (ANOVA) and Tukey’s test based on *p* < 0.05 were performed to determine the significance of the differences in response variables between strains.

## 3. Results and Discussion

### 3.1. Overexpression of Active PPTase to Endow B. subtilis 168 with the Ability to Synthesise Surfactin

**Figure 2:**
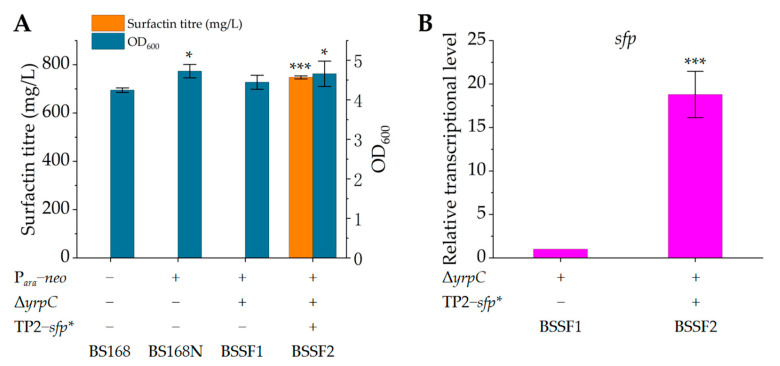
Surfactin titre and growth of wild-type *Bacillus subtilis* 168 (BS168), parent strain BS168N, *yrpC* gene knockout strain BSSF1 and *sfp* gene overexpression strain BSSF2, and the relative transcriptional levels of the sfp gene. (**A**) Surfactin titre and growth of the BS168, BS168N, BSSF1 and BSSF2 strains after 48 h of fermentation; (**B**) transcriptional level of *sfp* in strain BSSF2 relative to that in control strain BSSF1 (defined as 1). Significant differences were determined by SPSS statistics 17.0 based on *p* < 0.05 (*) and *p* < 0.001 (***).

PPTase encoded by sfp is responsible for connecting the long arm of coenzyme A (CoA), 4′-phosphopantothenamine, to the conserved serine residue in the peptidyl carrier protein (PCP), which converts PCP from an inactive apo-form to active holo-form, thereby initiating surfactin synthesis [41]. Due to the presence of an additional T_496_ base in the nucleotide sequence of *sfp* in *B. subtilis* 168, its transcription is terminated prematurely (Figure S1). Therefore, *sfp* is an inactive pseudogene in *B. subtilis* 168. Indeed, no surfactin was detected in the supernatant of its fermentation broth (Figure 2A and Figure S2A). To make the PPTase active, we designed primers to amplify the preceding and following nucleotide sequences of the *sfp* gene, removing the T_496_ base in the process. These two nucleotide sequences were spliced together through overlapping PCR, then spliced with the strong TP2 promoter [36] to integrate into the *yrpC* locus of the genome of strain BS168N, yielding recombinant strain BSSF2 (Table 1). In addition, we constructed *yrpC* gene knockout strain BSSF1 as a control for BSSF2. In *B. subtilis*, *yrpC* encodes glutamate racemase, which catalyses the racemisation of L-glutamate to produce D-glutamate [42]. L-glutamate is one of the precursors for synthesising surfactin, so the knockout of *yrpC* should reduce the consumption of L-glutamate. Moreover, Wang et al. [29] inhibited the expression of *yrpC* using CRISPRi technology, which indeed promoted surfactin synthesis.

After 48 h of fermentation, strain BSSF2 produced 747.5 ± 6.5 mg/L of surfactin (Figure 2A and Figure S2B). There were four components of the surfactin standards, corresponding to peaks 1, 2, 3 and 4 (Figure S2C,D). The differences between them were the amino acid residues at positions 2, 4 and 7 of the circular peptide (leucine, isoleucine or valine) and the length of the carbon chain (C13 to C15) [31]. The relative transcriptional level of sfp in BSSF2 was increased 17.8-fold relative to BSSF1 (Figure 2B). Compared to previous studies [21,29,30], overexpression of PPTase led to different levels of surfactin production, possibly due to the varying activities or expression levels of PPTases from different sources. The amino acid sequences of PPTase from *B. amyloliquefaciens* MT45 (GenBank: ASF27580.1), *B. amyloliquefaciens* DSM7 (GenBank: CBI41443.1), *B. velezensis* BS-37 (GenBank: AWG39808.1) and the active PPTase from *B. subtilis* 168 are not completely identical, as shown in the alignment (Figure S3). The above results indicated that PPTase was the key enzyme for *B. subtilis* to synthesise surfactin, and the recovery of endogenous PPTase activity endowed *B. subtilis* 168 with the ability to produce surfactin.

### 3.2. Effects of Plipastatin Synthetase and Phosphotransferase Deficiency on Surfactin Synthesis

**Figure 3:**
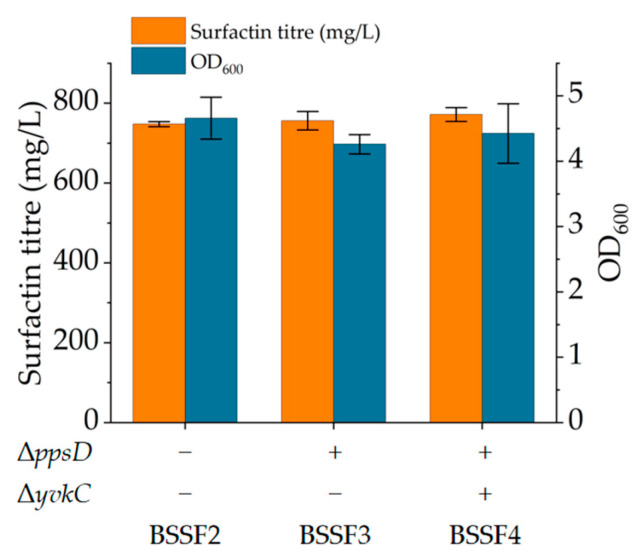
Surfactin titre and growth of control strain BSSF2, *pssD* gene knockout strain BSSF3 and *yvkC* gene knockout strain BSSF4 after 48 h of fermentation.

In *B. subtilis*, the *ppsABCDE* operon encodes plipastatin synthase that catalyses the synthesis of plipastatin from ten amino acids, including L-glutamate, D-alanine and L-tyrosine, along with hydroxyl fatty acids [43]. To increase the flow of amino acids and hydroxyl fatty acids towards surfactin synthesis, the *ppsD* gene in the *ppsABCDE* operon was knocked out, resulting in knockout strain BSSF3. The fermentation results showed that there was almost no change in surfactin production (Figure 3). Coutte et al. [44] inserted the spectacomycin resistance gene *spc* into *ppsA* to disrupt plipastatin synthesis in *B. subtilis*, resulting in a 80.7% increase in surfactin production after 24 h of fermentation in a Landy medium and a 2.9% decrease after 48 h of fermentation. The above results indicated that the effect of *pps* disruption on surfactin production was related to the fermentation medium and fermentation time.

In *B. subtilis* 168, YvkC may consume ATP and catalyse the synthesis of pyruvate to phosphoenolpyruvate. In addition, the *yvkC* gene encodes flavonoid phosphate synthetase, which catalyses the ATP-dependent phosphorylation of flavonoids to generate flavonoid monophosphates, AMP and orthophosphate [45]. To block gluconeogenesis and flavonoid monophosphate synthesis and reduce ATP consumption, the *yvkC* gene in the genome of BSSF3 was knocked out, resulting in knockout strain BSSF4. The fermentation results indicated that this had no significant positive effect on the synthesis of surfactin (Figure 3). Although surfactin synthesis requires ATP, the weaker activity of YvkC may result in less ATP consumption by its catalytic reaction and its knockout having no significant positive effect on surfactin synthesis.

### 3.3. Overexpression and Identification of a Surfactin Transporter to Enhance Surfactin Synthesis

Since surfactin accumulates excessively in cells, it may disrupt the integrity of cell membranes [31], thereby affecting normal cell function and even leading to cell death, which is obviously disadvantageous for the sustained synthesis of surfactin. Therefore, promoting surfactin secretion may improve surfactin production. In *B. subtilis* 168, *yerP* (also known as *swrC*) encodes a protein similar to the acriflavin resistance protein that is involved in surfactin efflux [46], and the *ycxA* gene, located downstream of the *srfA* operon, encodes a protein homologous to members of the major facilitator superfamily (MFS) [47]. In *Bacillus thuringiensis*, the *krsE* gene encodes an efflux protein involved in lipopeptide efflux [48]. The amino acid sequence of KrsE (GenBank: AIG20548.1) of *B. thuringiensis* was BLAST in the *B*. *subtilis* genome database, and it was found that YfiS shares the highest homology with KrsE (Figure S4). In *B. subtilis* 168, *yfiS* encodes a protein similar to multidrug resistance proteins.

**Figure 4:**
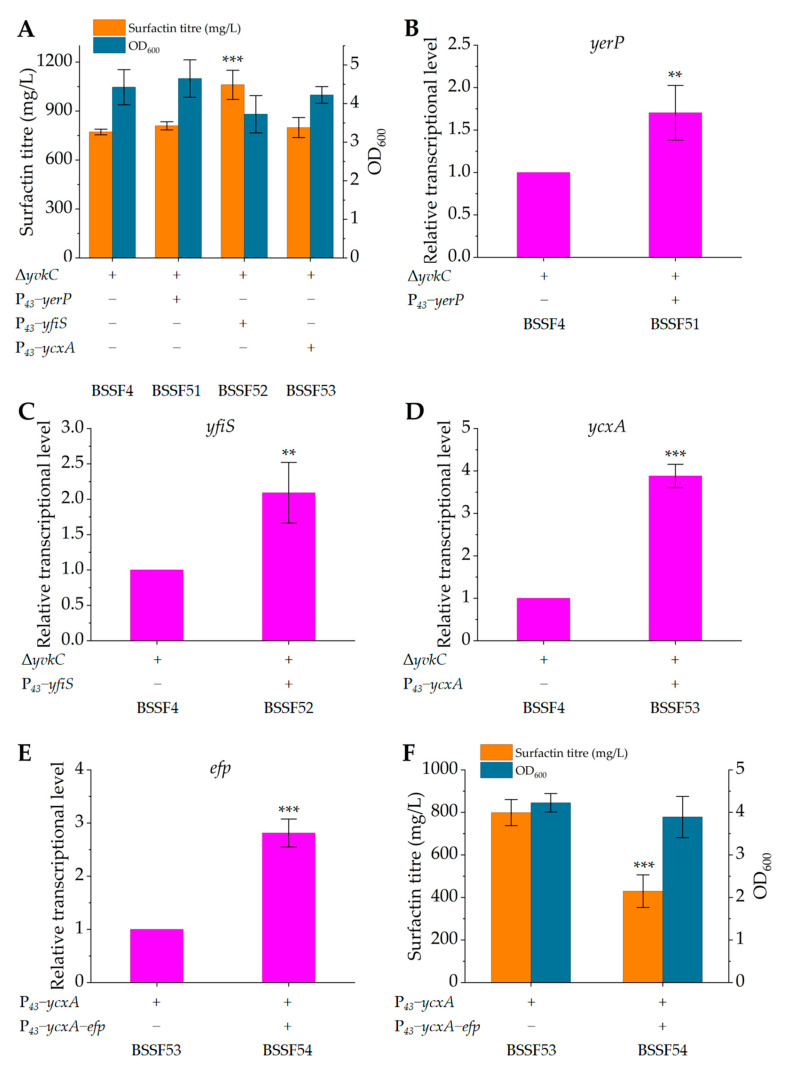
Surfactin titre, growth and relative transcriptional level of control strain BSSF4, and recombinant strains BSSF51 overexpressing *yerP*, BSSF52 overexpressing *yfiS*, BSSF53 overexpressing *ycxA* and BSSF54 overexpressing *ycxA-efp*. (**A**) Surfactin titre and growth of BSSF4, BSSF51, BSSF52 and BSSF53 after 48 h of fermentation; (**B**) transcriptional level of *yerP* in strain BSSF51 relative to that in control strain BSSF4 (defined as 1); (**C**) transcriptional level of *yfiS* in strain BSSF52 relative to that in strain BSSF4 (defined as 1); (**D**) transcriptional level of *ycxA* in strain BSSF53 relative to that in strain BSSF4 (defined as 1); (**E**) transcriptional level of *efp* in strain BSSF54 relative to that in control strain BSSF53 (defined as 1); (**F**) surfactin titre and growth of BSSF53 and BSSF54 after 48 h of fermentation. Significant differences were determined by SPSS statistics 17.0 based on *p* < 0.01 (**) and *p* < 0.001 (***).

The endogenous genes *yerP*, *yfiS* and *ycxA* were separately integrated into the *yvkC* locus of the BSSF3 genome under the control of the P*_43_* promoter [37], resulting in strains BSSF51, BSSF52 and BSSF53. After 48 h of fermentation, their surfactin production reached 809.3 ± 24.8 mg/L, 1060.7 ± 89.4 mg/L and 798.7 ± 61.4 mg/L, respectively, increased by 4.9%, 37.4% and 3.5%, respectively, compared to that of control strain BSSF4 (771.7 ± 17.3 mg/L; Figure 4A). Moreover, the growth of strain BSSF52 overexpressing *yfiS* was better than that of control strain BSSF4 and recombinant strains BSSF51 and BSSF53, during the first 24 h of fermentation (Figure S5C). The qRT-PCR results showed that adding one copy number to the genome resulted in a 0.70-fold (Figure 4B), 1.09-fold (Figure 4C) and 2.88-fold (Figure 4D) increase in the relative transcriptional levels of mRNAs of genes *yerP*, *yfiS* and *ycxA*, respectively. Overexpressing *ycxA* resulted in the largest increase in the transcriptional level, but its promotion effect on surfactin synthesis was the weakest. Considering that the translation of *ycxA* is likely to require elongation factor P (Efp) due to the presence of several consecutive proline residues [49], we constructed artificial operon P*_43_*-*ycxA-efp* and integrated it into the *yvkC* locus of the BSSF3 genome to obtain strain BSSF54. Although the relative transcriptional level of *efp* was increased by 1.81-fold (Figure 4E), co-expression of *ycxA* and *efp* resulted in a 46.3% decrease in surfactin production compared to control strain BSSF53 overexpressing *ycxA* alone (Figure 4F) and a slight decrease in strain growth (Figure S5D). The above results indicated that YfiS was mainly responsible for surfactin efflux under the culture conditions of this study.

### 3.4. Enhancing Transcription of the srfA Operon to Promote Surfactin Synthesis

**Figure 5:**
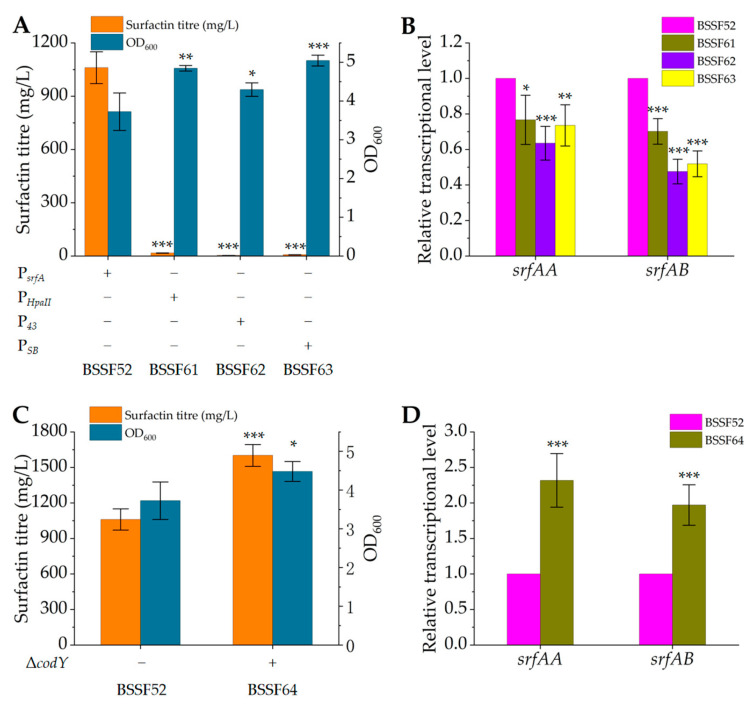
Surfactin titre, growth and relative transcriptional level of control strain BSSF52 and strains BSSF61, BSSF62 and BSSF63 obtained by replacing the native promoter of the *srfA* operon with promoters P*_HpaII_*, P*_43_* and P*_SB_*, respectively, and *codY* knockout strain BSSF64. (**A**) Surfactin titre and growth of BSSF52, BSSF61, BSSF62 and BSSF63 after 48 h of fermentation; (**B**) transcriptional levels of *srfAA* and *srfAB* in strains BSSF61, BSSF62 and BSSF63 relative to those in BSSF52 (defined as 1); (**C**) surfactin titre and growth of BSSF52 and BSSF64 after 48 h of fermentation; (**D**) transcriptional levels of *srfAA* and *srfAB* in strain BSSF64 relative to those in strain BSSF52 (defined as 1). Significant differences were determined by SPSS statistics 17.0 based on *p* < 0.05 (*), *p* < 0.01 (**) and *p* < 0.001 (***).

The nucleotide sequence of the *srfA* operon is up to 27 kb, making it difficult to increase its transcriptional level through overexpression. However, the promoter can determine the gene transcriptional level, so we attempted replacing the native promoter of the *srfA* operon. To this end, we used constitutive promoters P*_HpaII_*, P*_43_* and P*_SB_* [38] to replace the native promoter of the *srfA* operon in the genome of strain BSSF52, resulting in recombinant strains BSSF61, BSSF62 and BSSF63. The fermentation results showed that the surfactin production was only 16.5 ± 1.3 mg/L, 3.1 ± 0.2 mg/L and 7.1 ± 0.1 mg/L, respectively (Figure 5A), decreased by 98.4%, 99.7% and 99.3% compared to BSSF52. The qRT-PCR results showed that the relative transcriptional levels of *srfAA* and *srfAB* in the srfA operon decreased by varying degrees (Figure 5B). This was consistent with the previous research by Willenbacher et al. [50] and Jiao et al. [51]. The surfactin production of *B. subtilis* DSM 10T was decreased to 0.04 g/L from 0.62 g/L when the P*_veg_* promoter replaced the promoter of the *srfA* operon [50]. The ability of *B. subtilis* THY-7 to synthesise surfactin was lost when the P*_groE_* promoter induced by L-arabinose was used as a replacement [51]. Therefore, the initial transcription effect of these three promoters was not as strong as that of the native promoter of the *srfA* operon.

The global regulatory factor CodY recognises GTP and branched-chain amino acids (BCAAs) as specific signals and affects the expression of >100 genes associated with metabolism [52]. CodY binds to the promoter of the *srfA* operon, occupying the binding site between DNA and positive regulatory proteins, leading to inhibition of the transcription of this operon [53]. We knocked out the *codY* gene of strain BSSF52 to generate knockout strain BSSF64. Its surfactin titre was 1601.8 ± 91.9 mg/L (Figure 5C), 51% higher than that of control strain BSSF52, as in accordance with a previous report, in which this knockout resulted in a 17.3% increase [21]. The relative transcriptional levels of *srfAA* and *srfAB* increased by 1.31-fold and 0.97-fold, respectively (Figure 5D). This indicated that the knockout of *codY* could indeed alleviate the partial regulation of the *srfA* operon, thereby increasing its transcription and promoting the expression of surfactin synthase, significantly improving surfactin synthesis.

### 3.5. Increasing Surfactin Production through Fed-Batch Fermentation

**Figure 6:**
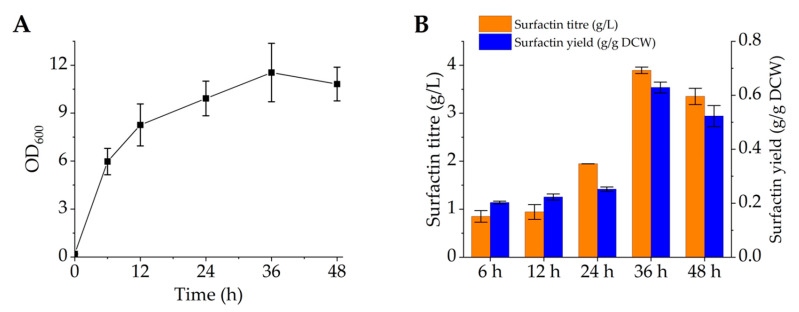
Growth curve, surfactin titre and yield of the final recombinant strain BSSF64 during fed-batch fermentation. (**A**) Growth curve of BSSF64 during fed-batch fermentations in a 5 L fermenter; (**B**) surfactin titre and yield of BSSF64 during fed-batch fermentations in a 5 L fermenter.

To improve the fermentation level of surfactin, we performed fed-batch fermentation of recombinant strain BSSF64 in a 5 L fermenter. After 6 h of fermentation, a large number of bubbles were evident, and the addition of soybean oil and polyether defoamer had no defoaming effect. Hence, fermentation could not proceed normally. A defoaming effect could only be achieved by adding organosilicon defoamer. After 36 h of fermentation, the maximum OD_600_ value of BSSF64 was 11.54 ± 1.83 (Figure 6A). Although its growth was almost twice that of flask fermentation, it was far below the growth achieved when using engineered *B. subtilis* for fed-batch fermentation to produce other high value-added chemicals [34,54]. The surfactin titres (yields) after fermentation for 12, 24, 36 and 48 h were 0.94 ± 0.15 g/L (0.22 ± 0.01 g/g DCW), 1.94 g/L (0.25 ± 0.01 g/g DCW), 3.89 ± 0.07 g/L (0.63 ± 0.02 g/g DCW) and 3.35 ± 0.17 g/L (0.52 ± 0.04 g/g DCW), respectively (Figure 6B). This might be due to inappropriate fermentation conditions leading to poor growth or to the toxicity of surfactin or organosilicon defoamer toward cells, which may have inhibited bacterial growth and surfactin synthesis. By optimising the fermentation medium and conditions alone, the OD_600_ of *B. subtilis* ATCC 21332 reached 60, and surfactin production was increased from 335 mg/L to 5.3 g/L [18]. Therefore, optimising the fermentation conditions, including carbon sources, nitrogen sources, pH, inoculum size and metal ions by adding Fe nanoparticles [55] or EDTA-Fe^2+^ [56], may be crucial for improving surfactin production in the future. Regarding the foaming phenomenon, a two-compartment biofilm bioreactor [57] or a foam trap [58] could be designed to avoid the addition of organosilicon defoamer.

## 4. Conclusions

The wild-type *B. subtilis* 168 strain cannot synthesise surfactin due to the presence of a frameshift mutation in the nucleotide sequence of *sfp* encoding PPTase, resulting in its inactivation. This study first removed the T_496_ base and integrated the overexpression of sfp*, which restored the activity of PPTase and increased the surfactin titre from zero to 747.5 ± 6.5 mg/L. The knockout of *ppsD* and *yvkC* had a minimal effect on surfactin synthesis. Overexpression of *yerP*, *yfiS*, *ycxA* and *ycxA-efp* indicated that YfiS might be the main surfactin transporter, and its overexpression increased surfactin production by 37.4% compared to the control strain. Replacing the native promoter of the *srfA* operon with three constitutive promoters significantly reduced the ability to synthesise surfactin, with yields ranging from 3.1 ± 0.2 mg/L to 16.5 ± 1.3 mg/L. However, the knockout of *codY* increased the surfactin titre by 51% to 1601.8 ± 91.9 mg/L. Finally, fed-batch fermentation was carried out in a 5 L fermenter without the need for antibiotics and inducers, and the highest titre of surfactin was 3.89 ± 0.07 g/L, with a yield of 0.63 ± 0.02 g/g DCW. Although the liquid production of surfactin was relatively low, production by dry weight was higher, indicating that engineered *B. subtilis* has the potential to become a cell factory for producing surfactin.

## Figures and Tables

**Table 1:** Bacterial strains and plasmids used in this study.

Name	Genotype	Source
Strain		
*Bacillus subtilis* 168 (BS168)	*trpC2*	Provided by Tianjin University
BS168N	trpC2, ΔaraR::P_ara_-neo	Provided by Tianjin University
BSD1-ΔyrpCm	BS168N, Δ*yrpC*::*cat-araR*, pHY300PLK-P*_43_*-*panD*	[33]
BSD1-ΔyvkCm	BS168N, Δ*yvkC*::*cat-araR*, pHY300PLK-P*_43_*-*panD*	[33]
BSΔ6-AD1m	BSΔ6, Δ*yvkC*::P*_43_*-*pfkA*::*cat-araR*, pHY300PLK-P*_43_*-*panD*	[33]
BSSF1	BS168N, Δ*yrpC*	This study
BSSF2	BS168N, Δ*yrpC*::TP2-sfp*	This study
BSSF3	BS168N, Δ*yrpC*::TP2-sfp*, Δ*ppsD*	This study
BSSF4	BS168N, Δ*yrpC*::TP2-sfp*, Δ*ppsD*, Δ*yvkC*	This study
BSSF51	BS168N, Δ*yrpC*::TP2-sfp*, Δ*ppsD*, Δ*yvkC*::P*_43_*-*yerP*	This study
BSSF51m	BS168N, ΔyrpC::TP2-sfp*, ΔppsD, ΔyvkC::P_43_-yerP::cat-araR	This study
BSSF52	BS168N, Δ*yrpC*::TP2-sfp*, Δ*ppsD*, Δ*yvkC*::P*_43_*-*yfiS*	This study
BSSF53	BS168N, Δ*yrpC*::TP2-sfp*, Δ*ppsD*, Δ*yvkC*::P*_43_*-*ycxA*	This study
BSSF53m	BS168N, ΔyrpC::TP2-sfp*, ΔppsD, ΔyvkC::P_43_-ycxA::cat-araR	This study
BSSF54	BS168N, Δ*yrpC*::TP2-sfp*, Δ*ppsD*, Δ*yvkC*::P*_43_*-*ycxA*-*efp*	This study
BSSF61	BS168N, ΔyrpC::TP2-sfp*, ΔppsD, ΔyvkC::P_43_-yfiS, P_HpaII_-srfA	This study
BSSF62	BS168N, Δ*yrpC*::TP2-sfp*, Δ*ppsD*, Δ*yvkC*::P*_43_*-*yfiS*, P*_43_*-*srfA*	This study
BSSF63	BS168N, Δ*yrpC*::TP2-sfp*, Δ*ppsD*, Δ*yvkC*::P*_43_*-*yfiS*, P*_SB_*-*srfA*	This study
BSSF64	BS168N, Δ*yrpC*::TP2-sfp*, Δ*ppsD*, Δ*yvkC*::P*_43_*-*yfiS*, Δ*codY*	This study
Plasmid		
pUC57-simple-VHb	Amp^R^, containing the constitutive promoter TP2 expression cassette	[34]
pMA5	Amp^R^, containing the constitutive P*_HpaII_* promoter	Laboratory stock
pUC57-simple-PyrG^E156K^	Amp^R^, containing the constitutive promoter P*_SB_* expression cassette	[34]

Amp^R^, ampicillin resistance; *, base T_496_ in the *sfp* gene sequence was removed.

**Table 2:** Sequences of the primers used in this study.

Primer	Sequence (5′→3′)
Knockout of the *yrpC* gene	
yrpC-U1	CTTACGCCAGACCTCCTA
yrpC-G2	ATCCTAACACAATCCTTCCAT
yrpC-D2	GTTGCCTGAGACTGTTACT
Overexpression of the sfp* gene	
yrpC-U2q	ACCATCAACGCAACCATAAACTCGCATCCTATCAATGTGA
TP2-1	GTTTATGGTTGCGTTGATGG
TP2-2	CATTCTTTACCCTCTCCTTTTAA
sfp1-1q	TTAAAAGGAGAGGGTAAAGAATGAAGATTTACGGAATTTATATGGAC
sfp1-2	TGAAAAGGAATCAGCGGAAG
sfp2-1	CTTCCGCTGATTCCTTTTCA
sfp2-2	ACTGTTGATGAGCCATTTATA
yrpC-D1q	TATAAATGGCTCATCAACAGTAACGGGCTCAATCACCTT
Knockout of the *ppsD* gene	
ppsD-U1	TTGCTCATCCGACTGTTG
ppsD-U2	GCAGTTCCATATTCTGAAGG
ppsD-D1q	TCCTTCAGAATATGGAACTGCTGTTGCCAGAGGTTATTTGA
ppsD-D2	AATAGGTGCCGCTCATCT
ppsD-CR1q	CAGATGAGCGGCACCTATTTCTTCAACTAAAGCACCCAT
ppsD-CR2	TTATTCATTCAGTTTTCGTG
ppsD-G1q	CGCACGAAAACTGAATGAATAATTGAACGAACAGGCTACC
ppsD-G2	GCATCCGCTGATTCTGAT
Knockout of the *yvkC* gene	
yvkC-U1	CAATGGCTTTCGGCTGAT
yvkC-G2	TCTCCTTGAATGTCCTGATAC
yvkC-D2	AGTGGAGACGGTGAATGA
Overexpression of genes *yerP*, *yfiS*, *ycxA* and *ycxA-efp*	
yvkC-P2	TTGTAAATTCCTCTCTTACCTAT
yerP-1q	TATAGGTAAGAGAGGAATTTACACATGACCAGTCAGTCAATAAAAA
yerP-2	GCAGACCAGACAACGAAT
yvkC-yerP-D1q	CATTCGTTGTCTGGTCTGCTGCTGTGATAGAGGATGAA
yvkC-yerP-G2	TGTCCTGATACATCGCTTG
CX-P43-1	ATACAGCCTTTGAACATACG
CX-yerP-2	ATCACAACGATGGAGTCAT
CX-yerP-3	GCCATCTTCGGTGCTATT
CX-yerP-5	AAGCGAAAGAACACAAACC
yfiS-1q	TATAGGTAAGAGAGGAATTTACAAATGGAAAAACCGTTGTTTCG
yfiS-2	ACATCCTTCATCGTCGTTAA
yvkC-yfiS-D1q	ATTAACGACGATGAAGGATGTATGAAGTATTGGCGAAGTTC
yvkC-yfiS-G2	GGGAGGTATGTGTGATTGAT
ycxA-1q	TATAGGTAAGAGAGGAATTTACAAATGCGCACGTCTCCCAGGT
ycxA-2	CATATACACTGAACCAAGAAGG
ycxA-D1q	TCCTTCTTGGTTCAGTGTATATGATTCGTTGTCTGGTCTGC
2-ycxA-2	TTTTATATTGAATGGTGGGTTTCT
efp-1q	AAGAAACCCACCATTCAATATAAAATGTGATTGGAATATAGGAGGAC
efp-2	GCTTGCTGAAGTAGTCTTGT
yvkC-efp-D1q	GACAAGACTACTTCAGCAAGCATTCCTTCGTGGTTCAGTGT
CX-efp1	TTACTGATTGTCGCTGTGT
In situ substitution of promoter of *srfA* operon	
srf-U1	GAGTTATCCTTGGACAATCAG
srf-U2	ACTGCTGCGTTGAATCTT
srf-C1q	AAAGATTCAACGCAGCAGTTCATCAAGTAAAGCACCCAT
srf-C2	ACAGTCGGCATTATCACATA
srf-P1-1q	ATATGTGATAATGCCGACTGTAATACTTCCTGTCCCTTGCT
srf-P1-2	TTGTAAATCGCTCCTTTTTAGG
srf-D1q	CCTAAAAAGGAGCGATTTACAAATGGAAATAACTTTTTACCCTTTAAC
srf-D2	CCGTCACAACATCATTCTG
CX-P1-1	AATACTTCCTGTCCCTTGCT
2-srf-C2	ACAGTCGGCATTATCACTTA
srf-P2-1q	ATAAGTGATAATGCCGACTGTATTCAGCCATAGAACATACG
srf-P2-2	TTGTAAATTCCTCTCTTTCCTAT
2-srf-D1q	TATAGGAAAGAGAGGAATTTACAAATGGAAATAACTTTTTACCCTTTAAC
CX-P2-1	ATTCAGCCATAGAACATACG
srf-P3-1q	ATAAGTGATAATGCCGACTGTAAAACGAAGAGAGAACATAGTAG
srf-P3-2	TTTGAAATCCTCCTTTTGTCC
3-srf-D1q	GGACAAAAGGAGGATTTCAAAATGGAAATAACTTTTTACCCATTAAC
CX-P3-1	ACCCATTATTACAGCAGGAA
Knockout of the *codY* gene	
codY-U1	GAGACTTCTGTTCGGCTTAT
codY-U2	ACCTCCTAAACATTCCTCAT
codY-D1q	TATGAGGAATGTTTAGGAGGTGCTTTATTTGCTGGGTTGAA
codY-D2	TATGATCTAGTGCTGCTGAC
codY-CR1q	TGTCAGCAGCACTAGATCATAACTTCAACTAAAGCACCCAT
codY-CR2	GTCTTCTTCCACCACTTG
codY-G1q	TCAAGTGGTGGAAGAAGACGGTAAACTACAAGGAAATGG
codY-G2	TTCTGAGTGCGTTCACAATA
Quantitative RT-PCR	
RT-ccpA1	ACGAGCATGTGGCGGAATT
RT-ccpA2	CGATAGCGACTGACGGTGTT
RT-sfp1	ATAAGCAGGCAGTATCAGTT
RT-sfp2	CGGAGTGAGAAATGTTGAAA
RT-yerP1	ATGACTCCATCGTTGTGAT
RT-yerP2	ATTTCCTTCGTCGCTTCA
RT-yfiS1	TTCTTTCTTTCCGCTGTCA
RT-yfiS2	TAGAAGTAAGTGCTGCTGTT
RT-ycxA1	GCAGAGCACCTATACCATT
RT-ycxA2	ACGCCGAAGTACAGGATA
RT-efp1	GGATGAAACACTTGGTATCG
RT-efp2	CTGACGCTGTATCACCTT
RT-srfAA1	GGTCAGCAATACGGAAGTA
RT-srfAA2	TCTGGACGGTTGTAATAGC
RT-srfAB1	GCTCCATATCGTCCAGAAG
RT-srfAB2	GGCGGTGTTCACTATTGT

*, base T496 in the *sfp* gene sequence was removed.

## Data Availability

All data are contained within the article and the Supplementary Materials.

## Supplementary Materials

The following supporting information can be downloaded at https://www.mdpi.com/article/10.3390/microorganisms12050998/s1: Figure S1: Nucleotide sequences of the *sfp* gene in *Bacillus subtilis*. (**A**) Nucleotide sequence of the inactive pseudogene *sfp* in wild-type *B. subtilis* 168. (**B**) Nucleotide sequence of the active *sfp* gene; Figure S2: HPLC profiles of the samples and standards. (**A**) HPLC profile of the supernatant of wild-type *B. subtilis* 168 fermented for 48 h, concentrated 4-fold after treatment. (**B**) HPLC profile of the supernatant of recombinant strain BSSF2 fermented for 48 h, concentrated 4-fold after treatment. (**C**) HPLC profile of the 0.2 g/L surfactin standard. (**D**) HPLC profile of the 5.0 g/L surfactin standard; Figure S3: Alignment results of the amino acid sequences of PPTase from different sources. GenBank: ASF27580.1, PPTase from *Bacillus amyloliquefaciens* MT45; GenBank: CBI41443.1, PPTase from *Bacillus amyloliquefaciens* DSM7; GenBank: AWG39808.1, PPTase from *Bacillus velezensis* BS-37 DSM7; GenBank: P39135.2, active PPTase from *Bacillus subtilis* 168; Figure S4: Alignment results of the amino acid sequences of KrsE from *Bacillus thuringiensis* (GenBank: AIG20548.1) and YfiS of *B. subtilis* 168 (GenBank: CAL0278229.1); Figure S5: Effects of different gene modifications on bacterial growth. (**A**) Growth curves of wild-type *Bacillus subtilis* 168 (BS168), parental strain BS168N, *yrpC* gene knockout strain BSSF1 and *sfp* gene overexpression strain BSSF2. (**B**) Growth curves of control strain BSSF2, *pssD* gene knockout strain BSSF3 and *yvkC* gene knockout strain BSSF4. (**C**) Growth curves of control strain BSSF4, *yerP* gene overexpression strain BSSF51, *yfiS* gene overexpression strain BSSF52 and *ycxA* gene overexpression strain BSSF53. (**D**) Growth curves of control strain BSSF53 and *ycxA-efp* gene overexpression strain BSSF54. (**E**) Growth curves of control strain BSSF52 and strains BSSF61, BSSF62 and BSSF63 obtained by replacing the native promoter of the *srfA* operon with promoters P*_HpaII_*, P*_43_* and P*_SB_*, respectively. (**F**) Growth curves of control strain BSSF52 and *codY* gene knockout strain BSSF64.
