# Supplementary material for: Improving Surfactin Production in Bacillus subtilis 168 by Metabolic Engineering
Source: Microorganisms. 2024 May 15;12(5):998. doi: 10.3390/microorganisms12050998 (PMC11124408; doi:10.3390/microorganisms12050998)
Supplement: Supplementary file 1 [file microorganisms-12-00998-s001.zip › microorganisms-2999573-supplementary.pdf]

Supplementary Materials

Improving Surfactin Production in *Bacillus subtilis* 168 by Metabolic Engineering

Zihao Guo [1], Jiuyu Sun [1], Qinyuan Ma [1], Mengqi Li [1], Yamin Dou [1], Shaomei Yang [1,*], Xiuzhen Gao [1,*]

[1] *School of Life Sciences and Medicine, Shandong University of Technology, 266 Xincun West Road, Zibo 255049, China*

[*] Correspondence: shaomei@sdut.edu.cn; gaoxz@sdut.edu.cn

A

ATGAAGATTTACGGAATTTATATGGACCGCCCGCTTTCACAGGAAGAAAATGAACGGTTCATGTCTTTCATATC
ACCTGAAAAACGGGAGAAATGCCGGAGATTTTATCATAAAGAAGATGCTCACCGCACCCTGCTGGGAGATGT
GCTCGTTCGCTCAGTCATAAGCAGGCAGTATCAGTTGGACAAATCCGATATCCGCTTTAGCACGCAGGAATAC
GGGAAGCCGTGCATCCCTGATCTTCCCGACGCTCATTTCAACATTTCTCACTCCGGACGCTGGGTCATTTGCGC
GTTTGATTCACAGCCGATCGGCATAGATATCGAAAAAACGAAACCGATCAGCCTTGAGATCGCCAAGCGCTTC
TTTTCAAAAACAGAGTACAGCGACCTTTTAGCAAAAGACAAGGACGAGCAGACAGACTATTTTTATCATCTAT
GGTCAATGAAAGAAAGCTTTATCAAACAAGGAAGGCAAAGGCTTATCGCTTCCGCT**TGA**TTCCTTTTCAGTGC
GCCTGCACCAGGACGGACAAGTATCCATTGAGCTTCCGGACAGCCATTCCCCATGCTATATCAAAACGTATGA
GGTCGATCCCGGCTACAAAATGGCTGTATGCGCCGTACACCCTGATTTCCCCGAGGATATCACAATGGTCTCGT
ACGAAGAGCTTTTA**TAA**

B

ATGAAGATTTACGGAATTTATATGGACCGCCCGCTTTCACAGGAAGAAAATGAACGGTTCATGTCTTTCATATC
ACCTGAAAAACGGGAGAAATGCCGGAGATTTTATCATAAAGAAGATGCTCACCGCACCCTGCTGGGAGATGT
GCTCGTTCGCTCAGTCATAAGCAGGCAGTATCAGTTGGACAAATCCGATATCCGCTTTAGCACGCAGGAATAC
GGGAAGCCGTGCATCCCTGATCTTCCCGACGCTCATTTCAACATTTCTCACTCCGGACGCTGGGTCATTTGCGC
GTTTGATTCACAGCCGATCGGCATAGATATCGAAAAAACGAAACCGATCAGCCTTGAGATCGCCAAGCGCTTC
TTTTCAAAAACAGAGTACAGCGACCTTTTAGCAAAAGACAAGGACGAGCAGACAGACTATTTTTATCATCTAT
GGTCAATGAAAGAAAGCTTTATCAAACAAGGAAGGCAAAGGCTTATCGCTTCCGCT**GA**TTCCTTTTCAGTGCG
CCTGCACCAGGACGGACAAGTATCCATTGAGCTTCCGGACAGCCATTCCCCATGCTATATCAAAACGTATGAG
GTCGATCCCGGCTACAAAATGGCTGTATGCGCCGTACACCCTGATTTCCCCGAGGATATCACAATGGTCTCGTA
CGAAGAGCTTTTA**TAA**

Figure S1. Nucleotide sequences of the *sfp* gene in *Bacillus subtilis*. (**A**) Nucleotide sequence of the inactive pseudogene *sfp* in wild-type *B. subtilis* 168. (**B**) Nucleotide sequence of the active *sfp* gene.

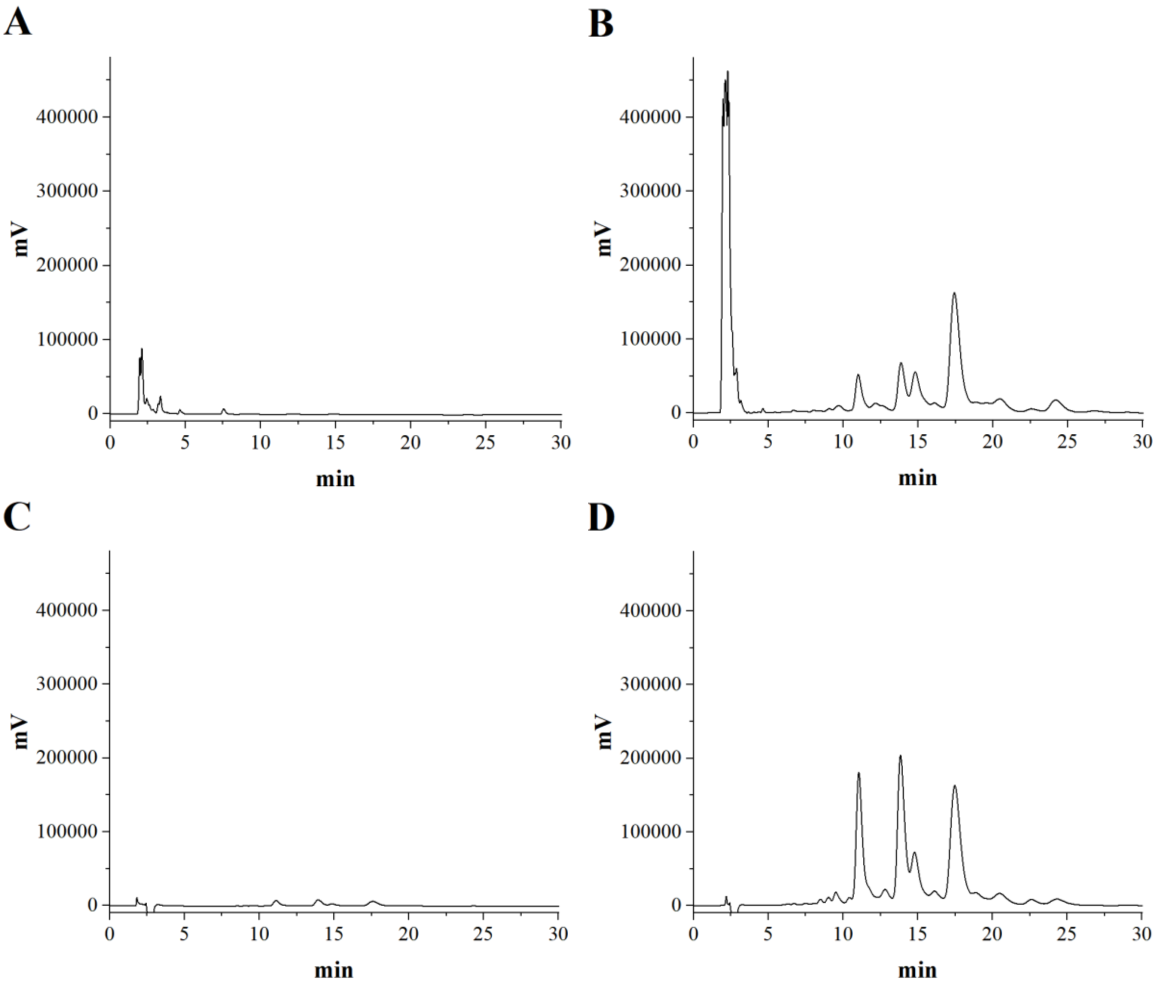

Figure S2. HPLC profiles of the samples and standards. (**A**) HPLC profile of the supernatant of wild-type *B. subtilis* 168 fermented for 48 h, concentrated 4-fold after treatment. (**B**) HPLC profile of the supernatant of recombinant strain BSSF2 fermented for 48 h, concentrated 4-fold after treatment. (**C**) HPLC profile of the 0.2 g/L surfactin standard. (**D**) HPLC profile of the 5.0 g/L surfactin standard.

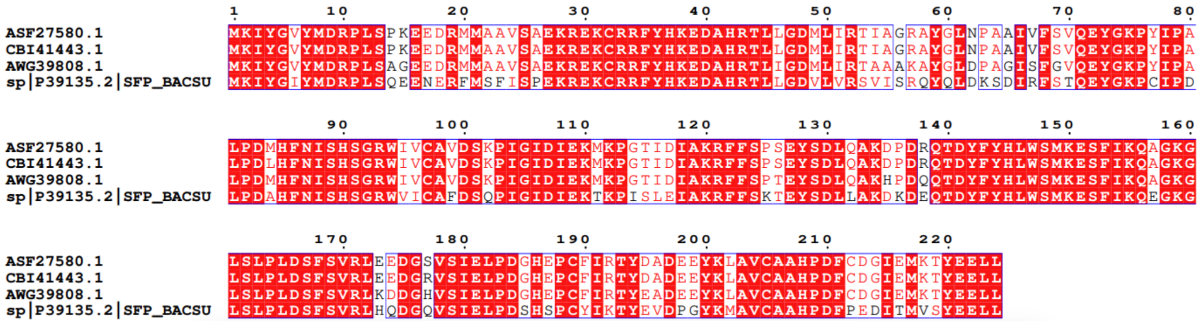

Figure S3. Alignment results of the amino acid sequences of PPTase from different sources. GenBank: ASF27580.1, PPTase from *Bacillus amyloliquefaciens* MT45; GenBank: CBI41443.1, PPTase from *Bacillus amyloliquefaciens* DSM7; GenBank: AWG39808.1, PPTase from *Bacillus velezensis* BS-37 DSM7; GenBank: P39135.2, active PPTase from *Bacillus subtilis* 168.

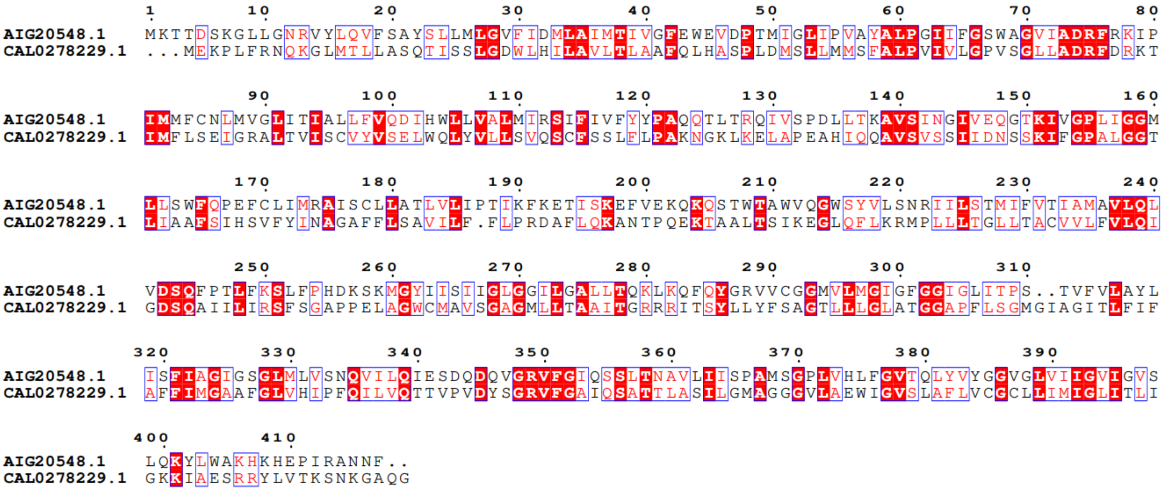

Figure S4. Alignment results of the amino acid sequences of KrsE from *Bacillus thuringiensis* (GenBank: AIG20548.1) and YfiS of *B. subtilis* 168 (GenBank: CAL0278229.1).

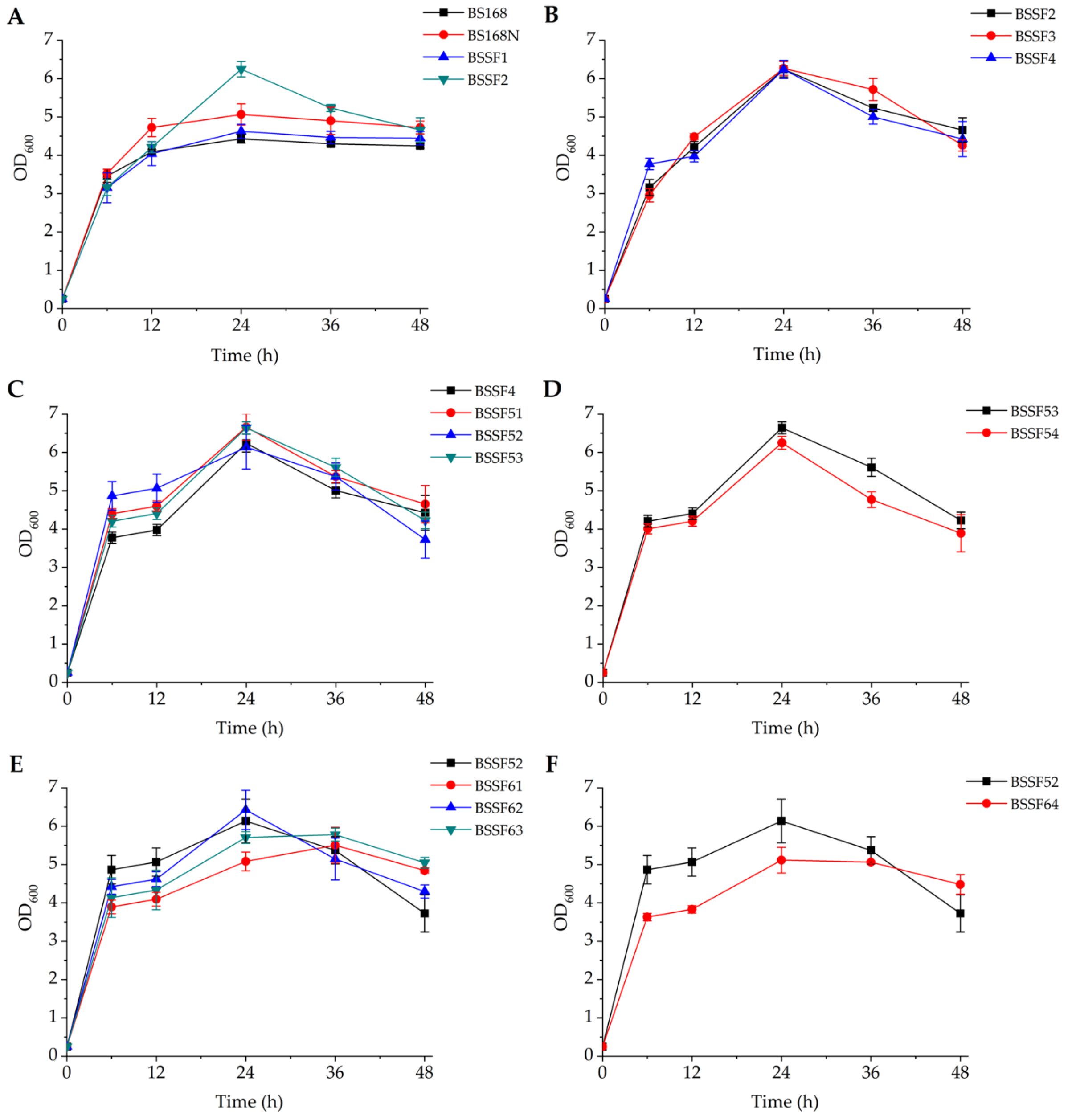

Figure S5. Effects of different gene modifications on bacterial growth. (**A**) Growth curves of wild-type *Bacillus subtilis* 168 (BS168), parental strain BS168N, *yrpC* gene knockout strain BSSF1 and *sfp* gene overexpression strain BSSF2. (**B**) Growth curves of control strain BSSF2, *pssD* gene knockout strain BSSF3 and *yvkC* gene knockout strain BSSF4. (**C**) Growth curves of control strain BSSF4, *yerP* gene overexpression strain BSSF51, *yfiS* gene overexpression strain BSSF52 and *ycxA* gene overexpression strain BSSF53. (**D**) Growth curves of control strain BSSF53 and *ycxA-efp* gene overexpression strain BSSF54. (**E**) Growth curves of control strain BSSF52 and strains BSSF61, BSSF62 and BSSF63 obtained by replacing

the native promoter of the *srfA* operon with promoters P_{HpaII}, P_{43} and P_{SB}, respectively. (**F**) Growth curves of control strain BSSF52 and *codY* gene knockout strain BSSF64.
